# Unusual prevalence of high-risk genotypes of human papillomavirus in a group of women with neoplastic lesions and cervical cancer from Central Mexico

**DOI:** 10.1371/journal.pone.0215222

**Published:** 2019-04-18

**Authors:** Rafael Gutiérrez Campos, Angélica Malacara Rosas, Elvia Gutiérrez Santillán, Mireya Delgado Gutiérrez, Rusland Enrique Torres Orozco, Elí Daniel García Martínez, Luis Fernando Torres Bernal, Alejandro Rosas Cabral

**Affiliations:** 1 Department of Chemistry, Center for Basic Sciences, Autonomous University of Aguascalientes, Aguascalientes, Aguascalientes, México; 2 Hospital General de Zona Número 6, Instituto Mexicano del Seguro Social, Monterrey, Nuevo León, México; 3 Hospital General de Zona Número 1, Instituto Mexicano del Seguro Social, Aguascalientes, Aguascalientes, México; 4 Department of Medicine, Center for Health Sciences, Autonomous University of Aguascalientes, Aguascalientes, México; Rudjer Boskovic Institute, CROATIA

## Abstract

Human papillomavirus has been identified as a main etiological agent in the development of cervical cancer. HPV 16 and 18 have been reported the most widely prevalent genotypes worldwide. We conducted a study analyzing the prevalence of high and low risk human papillomavirus viral types in the Mexican state of Aguascalientes and neighboring cities in the states of Jalisco and Zacatecas in central Mexico. Specific viral genotype was determined by a PCR and hybridization-based detection test. The presence of 37 high- and low-risk HPV genotypes was evaluated in 883 female participants. Of these, 350 presented low-grade squamous intraepithelial lesions (LGSIL), 176 presented high-grade squamous intraepithelial lesions (HGSIL), 107 suffered from cervical cancer and 250 women with negative cytological report for intraepithelial lesion or malignancy (NILM). HPV 51 was the most prevalent genotype, followed by HPV 16: overall prevalence of HPV 51, including single infections and co-infections was 31.2% in women with LGSIL, whereas prevalence of HPV 16 was 25.1%. Among women with HGSIL, HPV 51 prevalence was 47.2% and HPV 16 was 30.1%. Prevalence of HPV 51 in women with cervical cancer was 49.5% and type 16 was 33.6%. Between single and co-infections, most co-infections were not associated with later stages of the disease, except 51/16 and some others. HPV 51 showed a significant correlation with the progression of the disease (OR = 10.81 for LGSIL, 19.38 for HGSIL and 22.95 for ICC), and when analyzing all other genotypes, five different groups depending on their correlation with all lesion grades were determined. According to our findings, HPV genotype 51 has a higher prevalence than HPV 16 and 18 in the Mexican state of Aguascalientes and neighboring cities in the states of Jalisco and Zacatecas in Central Mexico.

## Background

Cervical cancer is the fourth leading cause of death by cancer in women worldwide. An estimated 500,000 new cases occur annually worldwide, and approximately 270,000 women die due to this illness each year [1]. Most of these cases occur in Latin America [2] and the Caribbean, as well as in Africa [3] and Asia [4]. Mexico has the second place in cervical cancer only below the African continent [5].

It has been demonstrated that human papillomavirus (HPV) is the indispensable etiological agent in the onset of cervical cancer [6]. Neoplastic alterations of the squamous or columnar epithelial cells of the cervix progress from low-grade to high-grade and finally to the malignant lesions of invasive cervical cancer.

Abundant literature worldwide reports viral genotypes 16 and 18 as the most important both in prevalence and oncogenic potential [7–9], except in Asian countries such as Japan, Taiwan, and some countries in east Africa [9]. HPV 16 and 18 have also been reported as prevalent in some states within Mexico [10–11]. However, some studies worldwide also mention a heterogeneous geographical distribution of high-risk HPV genotypes [12].

The goal of this study was to determine the prevalence of high- and low-risk HPV genotypes in cervical scrapes from 883 Mexican from central Mexico with dysplastic and cervical cancer lesions.

## Methods

### Ethics statement

The protocol was designed as a retrospective cohort study; we considered the results of 883 women attended in the Dysplastic Clinics of Hospital General de Zona Número 1 from 2010 to 2013. The women were invited to participate, signed informed consent according to the recommendations of the ethics review boards of the Institute. The protocol for information analysis was designed according to the guidelines of the Declaration of Helsinki and to the Official Mexican Standard NOM-012-SSA3–2012 ensuring respect for all human beings and protect their health, their individual rights and confidentiality of personal information. This analysis protocol information was submitted for review and was approved by the Ethics Committee of the Instituto Mexicano del Seguro Social.

### Study population

In a case-control study, cervical smear samples from a group of 883 women from central México were analyzed. All samples were collected at the Hospital General de Zona Número 1 of the Instituto Mexicano del Seguro Social, from May 2010 to December 2013. The women were invited to participate, signed informed consent according to the recommendations of the ethics review boards of the Institute; for underage patients, consent was obtained from an accompanying parent or legal guardian at the moment of the examination. The first group included cervical smear samples from women that, through conventional Pap test, had a negative cytological report for intraepithelial lesion or malignancy (NILM).

Premalignant lesions were determined by histopathology and classified according to the Cervical Intraepithelial Neoplasia (CIN) nomenclature [13]. The second group were women with LGSIL (low-grade squamous intraepithelial lesion). The third group were women with HGSIL (high-grade squamous intraepithelial lesion) as proposed previously [14]. A fourth group consisted of women diagnosed for invasive cervical cancer (ICC).

### Sample collection

Two fresh swabs or one biopsy per patient were obtained for HPV detection and genotyping. Cervical samples were collected with a cytobrush during gynecological examinations. It was inserted into the endocervical canal, rotated for 3 to 5 full turns, and then placed into the transport medium (Preserv- Cyt solution; Hologic, Bedford, MA) and stored at 4°C until DNA extraction.

### DNA extraction and HPV screening

DNA extraction was carried out using AmpliLute Liquid Media Extraction Kit (Roche). All samples were first screened by conventional single-round PCR utilizing the following sets of primers independently: GP5/GP6 [15], MY09/MY11, and PGMY09/11 [16]. Those samples that were positive for HPV with any of the primer sets were genotyped as described below.

### HPV detection and genotyping

All samples from control women without cervical lesions, but positive with GP5/GP6, MY09/MY11, and PGMY09/11, and cervical samples diagnosed as precursor lesions or cervical cancers were genotyped directly by the Linear Array HPV Genotyping Test (Roche Molecular Diagnostics). The Linear Array HPV Genotyping Test is registered for use of detecting 37 high- and low-risk HPV genotypes, and includes genotypes 6, 11, 16, 18, 26, 31, 33, 35, 39, 40, 42, 45, 51, 52, 53, 54, 55 (HPV 44 subtype), 56, 58, 59, 61, 62, 64 (HPV 34 subtype), 66, 67, 68, 69, 70, 71, 72, 73 (MM9), 81, 82 (MM4), 83 (MM7), 84 (MM8), IS39 (HPV 84 variant), and CP6108 (HPV 89). In each sample, the human β-globin gene was amplified as an internal control. After the hybridization reaction, the strips were read visually using a reference guide. All procedures were carried out following the manufacturer’s instructions. For further validation, a collection of random samples equivalent to 5% of the total number of analyzed samples was genotyped independently using a multiplex PCR protocol described by Sotlar et al. [17].

### Statistical analysis

A database of results from the genotyping of each sample was constructed, which contained the age of the analyzed patient and the genotypes detected in the samples, reported as presence (positive) or absence (negative) of each studied genotype. The data were analyzed using the SPSS statistical software (v. 22.0), reporting the prevalence of each genotype per lesion grade, age group and number of simultaneous genotypes in one sample (co-infection). Pearson’s chi-squared test was used to test if the prevalence of infection increased by age, number of genotypes detected or presence of certain genotypes. Correlation tables were also obtained to analyze the interaction of high-risk genotypes in each group of patients, and logistic regression was used to calculate the odds ratio of progression of the lesion for each genotype both in mono- and co-infection. For all comparison and independence tests, a p-value lower than 0.05 was considered statistically significant.

## Results

A total of 883 samples of women aged between 15 and 71 years were analyzed. All samples were obtained from Hospital General de Zona Número 1 of the Instituto Mexicano del Seguro Social of Aguascalientes city. This Health facility receives patients of Aguascalientes and the neighboring states of Jalisco and Zacatecas (central México). 248 samples were obtained from women without a cytological report for intraepithelial neoplasia or malignancy (NILM), 349 samples corresponded to low-grade squamous intraepithelial lesions (LSIL), 176 samples to high-grade intraepithelial lesions, and 110 samples were shown to have cervical cancer (CC).

In our study, 36 viral genotypes of both high- and low-risk were identified in all groups in different combinations of 1, 2, 3, 4, 5 or 6 different genotypes per sample. To facilitate the statistical analysis, co-infections of 4, 5 and 6 genotypes were combined into a single group of 4+ detected genotypes.

Of the 883 samples analyzed, 699 (79.2%) were positive for at least one HPV genotype. Table 1 shows the distribution of HPV infections by age group, as both single and multiple HPV infections. 73.7% of all positive samples were shown to have a single HPV infection, while multiple infections were present in the remaining 26.3% of the positive samples. All negative samples were found in the NILM group. The prevalence was proven to significantly increase by age (single infections, p = 0.012; multiple infections, p < 0.001). When accounting for lesion grade, the highest prevalence of single infections was found among the LGSIL group (60.7%, significant), the number of two genotype co-infections was distributed among HGSIL and ICC groups (37.1% and 37.9% respectively, not different significantly between each other), but when advancing to 3 simultaneous infections only HGSIL (65.2%) but not ICC (13.0%) had a significant increase in the overall prevalence of infections.

**Table 1:** Number of genotypes detected in the studied patients, divided by age group.

	Number of genotypes detected					Total
	0	1	2	3	4+	
**Age (years)**						
**< 15**	3 (1.6)	0 (0.0)	0 (0.0)	0 (0.0)	0 (0.0)	3 (0.3)
**16–25**	81 (44.0)	103 (20.0)	13 (11.2)	3 (6.5)	5 (23.8)	205 (23.2)
**26–35**	58 (31.5)	191 (37.0)	41 (35.3)	12 (28.3)	9 (42.9)	312 (35.3)
**36–45**	34 (18.5)	127 (24.6)	26 (22.4)	12 (26.1)	6 (28.6)	205 (23.2)
**46–55**	8 (4.3)	55 (10.7)	25 (21.6)	12 (26.1)	0 (0.0)	100 (11.3)
**56–65**	0 (0.0)	35 (6.8)	9 (7.8)	3 (6.5)	1 (4.8)	48 (5.4)
**> 66**	0 (0.0)	5 (1.0)	2 (1.7)	3 (6.5)	0 (0.0)	10 (1.1)
**Lesion group**						
**NILM**	184 (100.0)	46 (8.9)	11 (9.5)	3 (6.5)	4 (19.0)	248 (28.1)
**LGSIL**	0 (0.0)	313 (60.7)	18 (15.5)	7 (15.2)	11 (52.4)	349 (39.5)
**HGSIL**	0 (0.0)	99 (19.2)	43 (37.1)	30 (65.2)	4 (19.0)	176 (19.9)
**ICC**	0 (0.0)	58 (11.2)	44 (37.9)	6 (13.0)	2 (9.5)	110 (12.5)

For each frequency, the percentage from all patients of the same age group was also obtained, and is indicated in brackets. A chi-squared independency test was performed to analyze if there was any difference among age groups (p < 0.05 considered statistically significant).

Next, the individual prevalence of each genotype per lesion grade was studied. It was found that several common high-risk genotypes were more prevalent among most of the lesion groups. The overall prevalence of HPV infection, defined as the presence of at least one HPV genotype among the studied, was 79.2%, while Table 2 shows the comparison of each of these genotypes per lesion group. It was found that genotypes 51, 33, 35, and 68 became more uniformly prevalent as the lesion progressed, while others like 16, 18, 31 and 66 were more prevalent upon reaching HGSIL, but then decreased in the ICC group.

**Table 2:** Prevalence of individual genotypes among all lesion groups.

Genotype	NILM	LGSIL	HGSIL	ICC	TOTAL
**6**	5 (2.0)	10 (2.8)	1 (0.6)	0 (0.0)	16 (1.8)
**11**	4 (1.7)	7 (2.0)	1 (0.6)	0 (0.0)	12 (1.4)
**16**	12 (4.9)	88 (25.2)	58 (33.0)	34 (30.9)	192 (21.8)
**18**	2 (0.8)	15 (4.3)	13 (7.4)	5 (4.6)	35 (4.0)
**26**	0 (0.0)	0 (0.0)	0 (0.0)	0 (0.0)	0 (0.0)
**31**	4 (1.7)	53 (15.2)	33 (18.8)	12 (10.9)	102 (11.6)
**33**	2 (0.8)	20 (5.8)	18 (10.2)	18 (16.4)	58 (6.6)
**35**	3 (1.2)	10 (2.9)	11 (6.3)	11 (10.0)	35 (4.0)
**39**	3 (1.2)	11 (3.2)	5 (2.9)	6 (5.5)	25 (2.8)
**40**	4 (1.6)	5 (1.4)	2 (1.1)	1 (0.9)	12 (1.4)
**42**	6 (2.4)	3 (0.9)	2 (1.1)	1 (0.9)	12 (1.4)
**45**	3 (1.2)	3 (0.9)	6 (3.4)	5 (4.6)	17 (1.9)
**51**	10 (4.0)	109 (31.2)	79 (44.9)	54 (49.1)	252 (28.6)
**52**	4 (1.7)	22 (6.3)	24 (13.7)	6 (5.5)	56 (6.3)
**53**	0 (0.0)	1 (0.3)	0 (0.0)	0 (0.0)	1 (0.1)
**54**	1 (0.4)	0 (0.0)	0 (0.0)	0 (0.0)	1 (0.1)
**55**	0 (0.0)	0 (0.0)	1 (0.6)	0 (0.0)	1 (0.1)
**56**	3 (1.2)	14 (4.0)	4 (2.3)	4 (3.7)	25 (2.8)
**58**	0 (0.0)	7 (2.0)	0 (0.0)	1 (0.9)	8 (0.9)
**59**	1 (0.4)	5 (1.4)	0 (0.0)	0 (0.0)	6 (0.7)
**61**	1 (0.4)	0 (0.0)	0 (0.0)	0 (0.0)	1 (0.1)
**62**	4 (1.7)	1 (0.3)	7 (4.0)	0 (0.0)	12 (1.4)
**64**	0 (0.0)	0 (0.0)	0 (0.0)	0 (0.0)	0 (0.0)
**66**	9 (3.7)	21 (6.0)	20 (11.4)	2 (1.8)	52 (5.9)
**68**	3 (1.2)	5 (1.4)	4 (2.3)	9 (8.2)	21 (2.4)
**69**	0 (0.0)	0 (0.0)	0 (0.0)	0 (0.0)	0 (0.0)
**70**	0 (0.0)	1 (0.3)	0 (0.0)	0 (0.0)	1 (0.1)
**71**	2 (0.8)	2 (0.6)	0 (0.0)	0 (0.0)	4 (0.5)
**72**	1 (0.4)	0 (0.0)	0 (0.0)	0 (0.0)	1 (0.1)
**73**	0 (0.0)	0 (0.0)	0 (0.0)	0 (0.0)	0 (0.0)
**81**	0 (0.0)	1 (0.3)	0 (0.0)	0 (0.0)	1 (0.1)
**82**	0 (0.0)	1 (0.3)	0 (0.0)	0 (0.0)	1 (0.1)
**83**	1 (0.4)	4 (1.2)	0 (0.0)	0 (0.0)	5 (0.6)
**84**	3 (1.2)	0 (0.0)	0 (0.0)	0 (0.0)	3 (0.3)
**IS39**	1 (0.4)	0 (0.0)	0 (0.0)	0 (0.0)	1 (0.1)
**CP6108**	3 (1.2)	0 (0.0)	0 (0.0)	0 (0.0)	3 (0.3)

The table shows the individual prevalence (in brackets) of all analyzed genotypes among lesion groups and in the whole studied population, to carry further analysis. Each percentage corresponds to the relative prevalence of the genotype in that lesion group compared to all positive results for that particular genotype in the whole population.

The individual odds ratios for the most prevalent high-risk genotypes were calculated for each lesion group, taking the NILM group as reference (Table 3). Once compared, it was found that while several genotypes showed a positive risk correlation with the development of the disease, only a handful sustained a significant increase of risk through every stage of development, only 33 and 51 had an increasing risk correlation as the lesion became more severe; others saw either a decrease in risk (e.g. 16 and 18) or no significant correlation in either earlier (e.g. 35) or later stages of the disease (like 52), but a significant one with other types of lesion.

**Table 3:** Odds ratio of progression of the lesion, compared to the NILM group.

Genotype	LGSIL		HGSIL		ICC	
	OR	P	OR	P	OR	P
**16**	**6.63** **(3.54–12.43)**	**< 0.001**	**9.67** **(5.00–18.70)**	**< 0.001**	**8.80** **(4.34–17.84)**	**< 0.001**
**18**	**5.52** **(1.25–24.38)**	**0.024**	**9.81** **(2.19–44.04)**	**0.003**	**5.86** **(1.12–30.67)**	**0.036**
**31**	**10.92** **(3.90–30.60)**	**< 0.001**	**14.08** **(4.89–40.55)**	**< 0.001**	**7.47** **(2.35–23.72)**	**0.001**
**33**	**7.48** **(1.73–32.29)**	**0.001**	**14.01** **(3.21–61.21)**	**< 0.001**	**24.07** **(5.48–105.75)**	**< 0.001**
**35**	2.41 (0.66–8.84)	0.184	**5.44** **(1.50–19.81)**	**0.010**	**9.07** **(2.48–33.22)**	**0.001**
**39**	2.66 (0.74–9.63)	0.135	2.39 (0.56–10.13)	0.238	**4.71** **(1.16–19.20)**	**0.030**
**45**	0.71 (0.14–3.54)	0.678	2.88 (0.71–11.68)	0.139	3.89 (0.91–16.57)	0.067
**51**	**10.81** **(5.52–21.17)**	**< 0.001**	**19.38** **(9.64–38.99)**	**< 0.001**	**22.95** **(11.01–47.86)**	**< 0.001**
**52**	**4.10** **(1.40–12.06)**	**0.010**	**9.63** **(3.28–28.30)**	**< 0.001**	3.52 (0.97–12.73)	0.055
**56**	3.41 (0.97–12.01)	0.056	1.90 (0.42–8.59)	0.404	3.08 (0.68–14.01)	0.145
**66**	**1.70** **(1.59–3.78)**	**0.016**	**3.41** **(1.51–7.67)**	**0.003**	0.49 (0.11–2.31)	0.358
**68**	1.19 (0.28–5.01)	0.813	1.90 (0.42–8.59)	0.404	**7.28** **(1.93–27.43)**	**0.003**

Each OR was calculated using ordinal logistic regression, including its IC of 95% (in brackets). A p-value lower than 0.05 is considered significant.

## Discussion

Cervical cancer is the second most frequent form of cancer in Mexico, with a standardized mortality rate between 2006 and 2010 of 9.2 deaths per 100,000 women, thus representing the second leading cause of death in Mexican women. Although a decrease on the frequency of cervical cancer has been reported in recent years, mainly because of the implementation of cytology-based detection programs since 1941, this strategy has not translated into a net reduction of mortality, kept excessively high and above the standardized rate per age group reported worldwide as 7.1 per 100,000 for this neoplasia [18].

Human papillomavirus (HPV) infection is the cause of almost the entirety of the reported cases of cervical cancer worldwide. The infection is considered the most frequent sexually transmitted disease in the world, with more than 80% of sexually active women becoming infected at least once in their lifetime with the virus [19] and it has been reported that its prevalence within populations depends on the increasing number of sexual partners. Generally, during the course of the infection, a clearance of the virus is carried on by the immune system; nonetheless, in an elevated proportion of cases an integration of the HPV genome into the host chromosomes can occur, with this event being key to viral oncogenesis, and therefore persistent high-risk HPV infections are considered as the principal cause of cervical cancer [20].

In our study–which included samples from patients with normal cytology, both low and high grade lesions, and cervical cancer–we found that 79.2% of the patients present an infection with at least one HPV genotype, a frequency slightly higher than reported by other authors both nationally and globally, in studies including all referred lesion groups (68.4% by [21], 67.7% by [22], 67.1% by [23], 64.5% by [24], 66.7% by [25] in South Africa, and 59.4% by [26] in Croatia to name a few), and lower than documented by the IARC (who report that the percentage of cases of cervical cancer potentially attributed to HPV infection worldwide is 86.9%), although this fraction varies widely according to the geographic area and its socio-economic level [27].

When considering the proportion of HPV infection according to the lesion grade, we can observe that 25.8% of the patients with normal cytology present the infection, either single or multiple, while the totality of patients with lesion development and cervical cancer present one or more HPV genotypes–higher than reported by others in relation to the lesion grade, like López-Rivera et al. who report 63.3% of positive results for HPV in low-grade lesions [10], but in concordance to Li et al. in cervical cancer [28]. This can be credited to the fact that all our patients are referred to colposcopy due to suspicion of malignant lesions and are not enrolled from an open population.

Regarding the frequency of single or multiple infections according to the lesion grade, we found that the prevalence of single infections was more elevated in low-grade lesions, which can indicate a dissipation effect of a determined genotype to achieve the integration of its genome to the host’s; while the exact mechanisms by which high-risk HPV genotypes succeed in the integration of their genome aren’t completely understood, it has been reported that with the development of next-generation sequencing it is possible to determine the exact integration site and the subsequent genomic rearrangements to it. It has been indicated that such phenomenon takes place through two main mechanisms, loop integration and direct integration, and that a better understanding of the integration sites might allow to understand those factors that give a competitive advantage to certain genotypes during the progression of the disease [29]. However, in our patients with high-grade lesions a synergic effect with even three different genotypes seems to happen, probably as a result of the expression of a higher viral load and even though conflict exists whether if the viral load size or the integration status are determinant factors for the development of high-grade lesions, in a study performed by Manawapat-Klopfer et al. with 664 Danish women it was demonstrated that a 10-fold increase of the viral load, in addition to a state of viral genome integration, was significantly associated to the presence of a high-grade lesion [30]. In the same way, Li et al. reported that the viral load of patients with multiple genotype infections is higher than the load of patients with single infections, and that such viral load is also dependent on the genotype itself, being higher for those belonging to the alpha-9 family [29]. HPV genotypes that most frequently infect the mucosa belong to the alpha-papillomavirus group and most of the high-risk genotypes involved with cancer development are found in the alpha-7 group (18, 39, 45, 59), alpha-9 (16, 31, 32, 33, 35, 52, 58) and alpha-5 (51) [30]. Moreover, Shen-Gunther et al. found through deep sequencing a similar frequency of multiple HPV infections (60%), and, when assessing the species’ diversity, demonstrated a loss of HPV genotype diversity and a dominance of a single genotype in high-grade lesions [31]. Consequently, when the severity of the lesion progresses the dominance of one genotype is higher, which accords with our findings that in only 22.5% of our studied patients with cervical cancer more than two genotypes were found.

HPV genotypes 16 and 18 are considered to cause approximately 70% of all cervical cancer cases worldwide, followed in order of frequency by genotypes 31, 33, 45, 52 and 58 [32–34]. Several authors in Mexico have reported that the most prevalent genotype is HPV 16, followed by 18, 33 and others, in accordance with reports across the world ([10] and [23] in Mexico City, [35] in Ciudad Juárez, [36] from Guadalajara, [37] in Nayarit); however, some other genotypes considered unusual have been found recently as the most prevalent, joined with different prevalence of HPV genotypes across the country. In our study, that comprised patients from Central Mexico, we found an unusually high prevalence of HPV 51 across all lesion grades (31.2% of low-grade lesions, 47.1% of high-grade lesions and 49.5% of cervical cancer cases) and also among samples of normal cytology (4.20%, only surpassed by HPV 16). In all these cases, genotype 51 is more commonly found among our study group than almost every other genotype frequently presented as highly prevalent both nationwide and worldwide–for example 18, whose prevalence among our samples was situated between 5-fold and 10-fold lower than HPV 51, particularly among cervical cancer cases. This atypical prevalence of HPV 51 has also been assessed by other studies in an emerging trend. That is the case of Jácome-Galarza et al., who report in a study of 159,288 women that genotype 59 is the most frequent (39.28%), followed by 51 (25.0%) and 45 (7.14%) [38]. Also, Gallegos-Bolaños et al. found in 1,163 women and 166 men that the most common infection was caused by HPV 51 (42%), then 52 (38%) and 16 (37%) [39], similar to our results. Similarly, Gultekin et al., in a study including a million Turkish women, reported that the most frequent HPV genotype detected was 16, followed by 51 and 31 [40]. Argyri et al. report HPV 16 as the most frequent, then 58 and 51 [41], and Kulhan et al. found in 11,624 Turkish women HPV 16 at 11.25%, HPV 31 at 7.83% and HPV 51 at 6.06% [42], meaning that in all these cases the prevalence of HPV 51 is at least more elevated than of HPV 18, commonly reported as the second most frequent genotype worldwide. It is also of interest among our findings that HPV 51 was found more commonly as a single infection than in combination with other genotypes, and even those multiple infections seems to decrease in significance as the lesion progresses, therefore indicating a persistence effect of HPV 51 that can lead to the development of more severe stages of the disease.

In this sense, Brancaccio et al. refer that the detection of HPV genotypes can vary widely depending mainly on the methodology used and the biological quantity of the sample [43], which can explain the differing prevalence found by us, due to the fact that each of the studied mentioned previously has used a different technique for the detection of HPV genome. Therefore, since HPV infection is population-specific, the findings among Western populations cannot be extrapolated to others, like Asians and probably Amerindians [44].

As previously mentioned, the existence of variability of HPV presence in Mexico is suggested from our data and other studies, and therefore it is important to completely define the geographic distribution of those specific HPV genotypes across different regions, in order to implement a successful early cervical cancer diagnosis program that allows to reduce the still elevated mortality rates which are still documented in the country [37].

HPV infection patterns can offer an unrecognized benefit besides genotyping, and must be considered in the clinical risk assessment of women with low-grade lesions [20], to prevent their progression since the presence of high-risk genotypes is associated to a big number of intraepithelial low-grade lesions.

According to Chen et al., the heterogeneity and phylogeny of HPV isolates indicate an independent evolutionary history for each genotype. They found that the non-coding regions of the virus were the most variable and the capsid regions mostly stable, which indicates that certain variants or sub-clones are geographically related [45]; this would require a reassessment of genotype distribution in standardized nationwide studies that include different regions in a representative way.

In respect to the individual prevalence of each genotype according to lesion grade, we observed a differential behavior, even within high-risk genotypes which are shown to increase as the lesion progresses; this is most relevant to HPV 16 genotype, which has been reported to increase its presence in direct relation to the lesion grade, even in a meta-analysis of 30,165 women with HPV infection [46], but different from our results which show that HPV 16 prevalence peaks at a high-grade lesion but then decreases when the lesion progresses to cancer. In the same way we found that a HPV 16–51 concomitant infection follows the same pattern, although we consider that this observation origins from an elevated prevalence of HPV 51 observed in our study, and which is corroborated by other authors like Gallegos-Bolaños et al., who report an elevated prevalence of co-infection between HPV 51 and 52 in the Mexican population [39]. Elsewhere, high prevalence of HPV 51 in high-grade lesions and developing cervical cancer have been found in co-infection with other common high-risk genotypes such as 16, 18 or 33, most likely due to the high prevalence of the aforementioned genotypes in their respective populations [47–49].

We also report a higher rate of co-infections than reported by other authors in Mexico in our country and in other countries. HPV co-infection is frequent in both men and women, but its clinical significance is still uncertain. Some authors consider that co-infection increases the risk of cervical cancer development and translates into a significantly lower cancer-specific survival and a greater tendency to develop metastatic disease [50].

This diversity could be related to the phylogenetic associations between different HPV species or with the interaction of different oncoproteins (E6, E7) with proteins involved in apoptosis (it has been reported that different genotypes differentially increase myc expression [51] [52]) or E6, which can suppress the transcription of TLR9 [53] or genes involved in interferon signaling (STAT), producing an inactivation state of the immune system and the inflammatory response associated to the presence of the viral infection [54].

## Conclusion

We found an elevated prevalence of infection by HPV 51, a high rate of co-infections between HPV 51 and 16 and a differential association between the risks of progression of the disease from the asymptomatic infection until the development of cervical cancer among the different high-risk HPV genotypes.

## Supporting information

S1 File Raw database. The full database containing the genotyping results of all samples analyzed in this study.
(XLSX)
